# Modeling Circadian Phototransduction: Quantitative Predictions of Psychophysical Data

**DOI:** 10.3389/fnins.2021.615322

**Published:** 2021-02-05

**Authors:** Mark S. Rea, Rohan Nagare, Mariana G. Figueiro

**Affiliations:** ^1^Lighting Research Center, Rensselaer Polytechnic Institute, Troy, NY, United States; ^2^Icahn School of Medicine at Mount Sinai, New York, NY, United States

**Keywords:** circadian light, circadian stimulus, phototransduction, melatonin suppression, light at night, non-image forming effects of light

## Abstract

A revised computational model of circadian phototransduction is presented. The first step was to characterize the spectral sensitivity of the retinal circuit using suppression of the synthesis of melatonin by the pineal gland at night as the outcome measure. From the spectral sensitivity, circadian light was defined. Circadian light, thereby rectifies any spectral power distribution into a single, instantaneous photometric quantity. The second step was to characterize the circuit’s response characteristic to different amounts of circadian light from threshold to saturation. By doing so a more complete instantaneous photometric quantity representing the circadian stimulus was defined in terms of both the spectral sensitivity and the response magnitude characteristic of the circadian phototransduction circuit. To validate the model of the circadian phototransduction circuit, it was necessary to augment the model to account for different durations of the circadian stimulus and distribution of the circadian stimulus across the retina. Two simple modifications to the model accounted for the duration and distribution of continuous light exposure during the early biological night. A companion paper (https://www.frontiersin.org/articles/10.3389/fnins.2020.615305/full) provides a neurophysiological foundation for the model parameters.

## Introduction

Circadian phototransduction is the process that converts optical radiation incident on the retina to neural signals reaching the suprachiasmatic nucleus (SCN). The circadian phototransduction mechanism can be conceived as a unique neural circuit in the retina with a spectral sensitivity to optical radiation and a response characteristic to different amounts of that optical radiation. Modeling human circadian phototransduction requires a systematic and converging approach to understand how a retinal circuit might perform this conversion. No model of circadian phototransduction can be justified by the results of a single experiment. Rather, such a model must be able to quantitatively characterize the photic stimulus incident on the retina such that the circadian system response can be accurately and consistently predicted. Moreover, any model of circadian phototransduction should be consistent with retinal neurophysiology and neuroanatomy. With regard to this last requirement, a companion paper (https://www.frontiersin.org/articles/10.3389/fnins.2020.615305/full) describes the neural foundation for the revised model of circadian phototransduction described here.

The present article is specifically aimed at establishing a quantitative measure of optical radiation incident on the human retina as it stimulates a neural circuit that, in turn, stimulates the SCN. In short, a system of photometry is proposed such that the photic circadian stimulus is quantified in terms of both spectrum and amount. To do so a functional relationship between the stimulus (spectrum and amount) and the response must be established experimentally and then validated by *a priori* hypothesis testing.

A model of circadian phototransduction must be based upon a measurable behavioral response resulting from photic stimulation of an intact, functional retina. Responses include light-induced nocturnal melatonin suppression and phase shifting as measured by changes in melatonin concentrations after dim light melatonin onset or changes in minimum core body temperature. As such, the circadian stimulus must be inferred from a downstream outcome measure. Nocturnal melatonin suppression is an excellent outcome measure for characterizing the spectral sensitivity and the response characteristic of the circadian phototransduction neural circuit stimulating the SCN because the primary, if not only, light-sensitive pathway to the pineal gland is from the SCN (Moore, 1996; Macchi and Bruce, 2004). Thus, light-induced nocturnal melatonin suppression gives a window into the otherwise unreachable SCN response to retinal light exposure. The ability to predict downstream behavioral responses from a circadian stimulus can, of course, be compromised because (a) the temporal characteristics of a phototransduction circuit and the distribution of these circuits across the retina are not defined by the circadian stimulus as a photometric quantity and (b) few, if any, behavioral response other than nocturnal melatonin suppression are so closely tied to the SCN response. Thus, the circadian stimulus, like other photometric quantities, is not a complete specification of the photic stimulus to the circadian system and is not necessarily the only determinate of a circadian system outcome (e.g., sleep, alertness, and cortisol concentration) (Rea et al., 2020).

## Modeling the Neural Circuit Spectral Sensitivity

**FIGURE 1:**
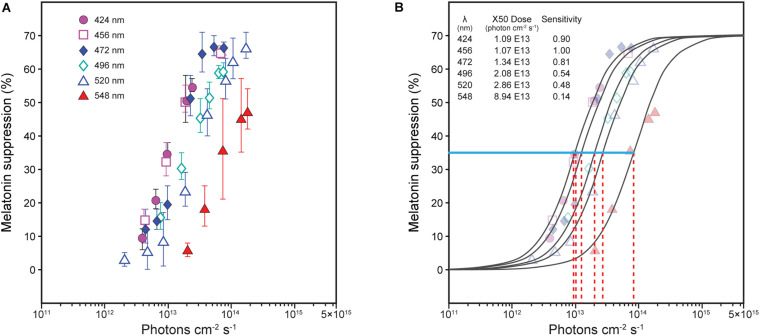
Nocturnal melatonin suppression for different narrowband spectra [**(A)**, Thapan et al. (2001)]. To determine spectral sensitivity, the amount of photon rate density (photons cm^–2^ s^–1^) needed at each wavelength [**(B)**, red dashed lines] to reach half-saturation [**(B)**, blue solid line, ≈ 35% suppression] is plotted relative to the wavelength requiring the least amount of energy to reach half-saturation (maximum sensitivity). Two sets of derived spectral sensitivity estimates using this procedure, both using irradiance rather than photon rate density at the eye were developed, one for Thapan et al. (2001) and one from Brainard et al. (2001), as shown in Figure 2.

**FIGURE 2:**
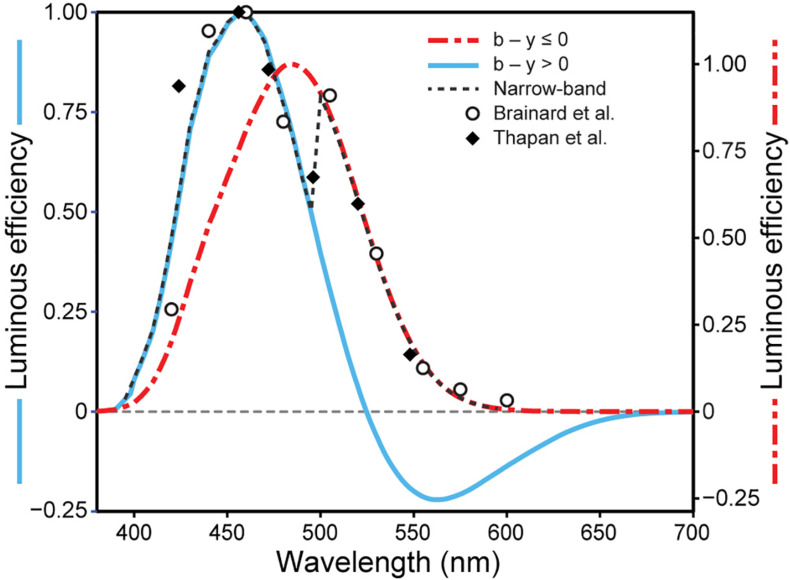
The relative sensitivity of different narrowband wavelengths for suppressing nocturnal melatonin from Brainard et al. (2001) and Thapan et al. (2001). Also shown are the predictions from the two-state circadian phototransduction model (Eq. 1), for narrowband (achromatic dashed line) and for “warm” polychromatic lights where b-y ≤ 0 (red dot/dash line) and for “cool” polychromatic lights where b-y > 0 (blue solid line) when the overall flux density on the retina is 300 scotopic lx (Rea and Figueiro, 2018).

Spectral sensitivity functions can be generated from data relating nocturnal melatonin suppression to log photon rate densities (photons cm^–2^ s^–1^) for each of a set of narrowband spectra (e.g., Figure 1A). A functional relationship is experimentally developed relating the stimulus magnitude (abscissa) to the response magnitude (ordinate) for all wavelengths (e.g., solid lines in Figure 1B). A constant criterion response is then established, usually the half-saturation value from the functional relationship (Figure 1B, blue solid line), and the amount of photon rate density (or irradiance) needed to produce that criterion response is determined for each wavelength (Figure 1B, red dashed lines). Maximum spectral sensitivity is associated with that wavelength needing the least amount of photon rate density (or irradiance) to reach the constant criterion response (Figure 1B, left-most red dashed line). Spectral sensitivity is then determined from the photon rate density (or irradiance) levels at each wavelength needed to reach the criterion response relative to the energy level needed for the most sensitive wavelength. Two spectral sensitivity estimates, using irradiance as the measure of optical radiation rather than photon rate density (Figure 1), of circadian phototransduction were developed based upon a constant criterion response methodology (Figure 2; Brainard et al., 2001; Thapan et al., 2001). The two spectral sensitivity estimates are very similar, with a peak sensitivity at or near 460 nm.

In 2005 a non-linear spectral sensitivity function for the human circadian system was proposed by Rea et al. (2005). This model was based, in part, on the first experiment (Figueiro et al., 2004) demonstrating that circadian phototransduction exhibited what is termed *subadditivity.* Subsequent experiments validated those results (Figueiro et al., 2005, 2008). For a subadditive system, different wavelengths interact to reduce the magnitude of the response to a light exposure that would be expected if the system simply integrated the energy at each wavelength (as with a luminous efficiency function). This observed phenomenon of subadditivity should not be surprising. Subadditivity would be expected if the neural circuit underlying circadian phototransduction included a spectral opponent color mechanism in the retina, either green versus red or blue versus yellow. In either case adding one wavelength of light to a longer wavelength of light (e.g., green with red or blue with yellow) will reduce the response of the spectral opponent neural channel.

To fit the melatonin suppression data (e.g., Figure 1B) and the spectral sensitivities (Figure 2), a spectral opponent blue versus yellow (b-y) color mechanism was part of the 2005 model. Specifically, a spectrally opponent S-ON bipolar neuron provides input to the intrinsically photosensitive retinal ganglion (ipRGC) neuron for light sources dominated by short wavelengths (“cool” light sources that would appear blue or have a bluish tint), but could not for light sources dominated by long wavelengths (“warm” light sources that would appear yellow or have a yellowish tint) [See companion paper (https://www.frontiersin.org/articles/10.3389/fnins.2020.615305/full) for details]. To predict spectral sensitivity for narrowband and polychromatic light sources, a two-state model was needed based upon the two response polarities of the b-y spectral opponent channel, blue *or* yellow. For “warm” sources (b-y ≤ 0), the spectral sensitivity of the circadian system was based upon the spectral sensitivity of the ipRGC alone. For “cool” sources (b-y > 0), spectral sensitivity was based upon the combined spectral sensitivities of the S-ON bipolar and that of the ipRGC. The modeled spectral sensitivity to “cool” sources included another form of non-linearity, a threshold, which was controlled by a light-level dependent, rod-cone interaction mechanism. The two-state equation underlying the 2005 model (Eq. 1) was postulated to characterize *circadian light*, abbreviated CL_*A*_, where the subscript “A” designates a numerical equivalence of CL_*A*_ = 1000 = 1000 (photopic) lx for CIE illuminant A (Rea et al., 2012) which is spectrally like a common incandescent light source. Predictions from the two-state model for narrowband spectra (achromatic dashed line) and for “warm” (red dot/dash line) and “cool” (blue solid line) polychromatic light sources at a given overall flux density on the retina (300 scotopic lx) were developed (Figure 2).


(1) $$C\,L_{A}=1548\{\int Mc_{\lambda }E_{\lambda }d\lambda +a_{b-y}\left(\int \frac{S_{\lambda }}{m\,p_{\lambda }}E_{\lambda }\text{d}\lambda -k\int \frac{V_{\lambda }}{m\,p_{\lambda }}E_{\lambda }\text{d}\lambda \right)-$$

$$a_{r\,o\,d}\left(1-e^{\frac{-\int V_{\lambda }^{{}^{\prime }}\,E_{\lambda }\,\text{d}\,\lambda }{R\,o\,d\,S\,a\,t}}\right)\},\text{b-y}>01548\int Mc_{\lambda }E_{\lambda }d\lambda \;,\text{b-y}\leq 0$$

where,


$$\begin{array}{c} b-y=\int \frac{S_{\lambda }}{m\,p_{\lambda }}\,E_{\lambda }\,\text{d}\,\lambda -0.2616\,\int \frac{V_{\lambda }}{m\,p_{\lambda }}\,E_{\lambda }\,\text{d}\,\lambda \end{array}$$

**Table:** 

*CL*_*A*_, circadian light.	*E*_λ_, light source spectral irradiance.
	*Mc*_λ_, melanopsin sensitivity (corrected for crystalline lens transmittance) (Wyszecki and Stiles, 1982).
*k* = 0.2616	*S*_λ_, S-cone fundamental (Smith and Pokorny, 1975).
*a*_*b–y*_ = 0.7	*mp*_λ_, macular pigment transmittance (Snodderly et al., 1984).
*a*_*rod*_ = 3.3	*V*_λ_, photopic luminous efficiency function (Commission Internationale de l’Éclairage, 1994).
*RodSat* = 6.5Wm^−2^	$V_{\lambda }^{\prime }$, scotopic luminous efficiency function (Commission Internationale de l’Éclairage, 1994).

## Modeling the Neural Circuit Response Characteristic

To model the response of the circadian phototransduction circuit to different amounts of optical radiation on the retina, the spectral sensitivity of the phototransduction circuit must be defined. Obviously, not every wavelength of optical radiation will be effective at evoking a circuit response (e.g., infrared or ultraviolet optical radiation). In other words, to establish a neural circuit response characteristic, all *effective* radiation must be considered simultaneously, specifically to account for subadditivity. The two-state, non-linear model of circadian light, CL_*A*_ in Eq. 1, was used for this purpose.

The sigmoidal logistic function in Eq. 2 was used to describe the response characteristic of the neural circuit underlying circadian phototransduction. The parameters in Eq. 2 were determined from mathematical modeling nocturnal melatonin suppression data from a variety of experiments using 1-h exposures to polychromatic lights (McIntyre et al., 1989; Rea et al., 2001, 2002; Figueiro et al., 2004). Since duration of exposure affects the amount of melatonin suppressed [e.g., McIntyre et al. (1989)], it was important to control this variable for modeling purposes. The nocturnal melatonin suppression data (e.g., Figure 1A) underlying the spectral sensitivities (Figure 2) were also included in the modeling exercise even though neither study used 1-h exposures; Thapan et al. (2001) used 0.5-h exposures while Brainard et al. (2001) used 1.5-h exposures. For spectral sensitivity estimates the duration of exposure is unimportant for the first 3 h of the biological night (Nagare et al., 2019c) because sensitivities at each wavelength are scaled relative to the maximum wavelength sensitivity within a given study. To use the absolute melatonin suppression data from both experiments, however, estimates of suppression following 1-h exposure were determined, first, by converting the optical radiation used in the Thapan et al. experiments from units of photon rate density to units of irradiance. Next, the Thapan and Brainard data were fitted with a sigmoidal logistic function similar that in Eq. 2. To estimate suppression in both experiments as if their observers had been given 1-h exposures, these fitted data sets were then shifted along the log irradiance abscissa until they were empirically aligned to a mid-point between the two sets of data. The magnitudes of the shifts were nearly proportional to the ratio of their different exposure durations. To a first approximation, this strategy should be valid because the response characteristic described by a sigmoidal function is fixed for any spectrum (e.g., Figure 1B) and should hold for a wide range of exposure durations (A more detailed discussion of this issue is included in a subsequent section). These transformed data were then used as part of the modeling exercise.

As the result, the sigmoidal function parameters become fixed for defining the *circadian stimulus*, abbreviated CS, following 1-h exposure. Importantly, for *any* set of stimulus conditions the neural circuit response characteristic (CS, Eq. 2) is assumed to be fixed *except* for the half-saturation constant (355.7 in Eq. 2). It should also be noted that CL_*A*_ can affect the half-saturation constant due to the spectral power distribution’s impact on rod-cone interactions affecting absolute threshold. Further, the half-saturation constant would be affected by stimulus conditions not included in the CL_*A*_ and CS formulations (e.g., exposure durations other than 1 h). With regard to this latter point, CS is not, therefore, a complete specification of the photic stimulus.


(2) $$C\,S=0.7*\left[1-\frac{1}{1+{\left(\frac{C\,L_{A}}{355.7}\right)}^{1.1026}}\right]$$

## Tests of the 2005 Model

As an introduction to testing the 2005 model predictions, it is worth reemphasizing the point made in the previous section that the operating characteristic of the neural circuit must be fixed once the circuit response exceeds threshold. See for example Figure 1A where the operating characteristic remains the same and only the half-saturation constant changes to account for circuit sensitivities to different wavelengths. Therefore, for testing model predictions it is inappropriate to allow parameters other than the half-saturation constant in the logistic function of Eq. 2 to vary in an attempt to improve the coefficient of determination for different sets of data [e.g., Brown (2020)]. It is also worth emphasizing that any test of a model must be able to predict circadian system response to *both* narrowband and broadband (polychromatic) light sources. Brown (2020), for example, could not fit both types of light sources. And as pointed out by Rea (2020), virtually any spectrally wide luminous efficiency that includes short wavelengths, including the “melanopsin only model” proposed by Brown (2020), can predict melatonin suppression for polychromatic light sources. The real test of a model must be based upon its ability to predict responses to any spectral power distribution, narrowband or broadband, and, of course must be consistent with the neuroanatomy and neurophysiology of the retina.

**FIGURE 3:**
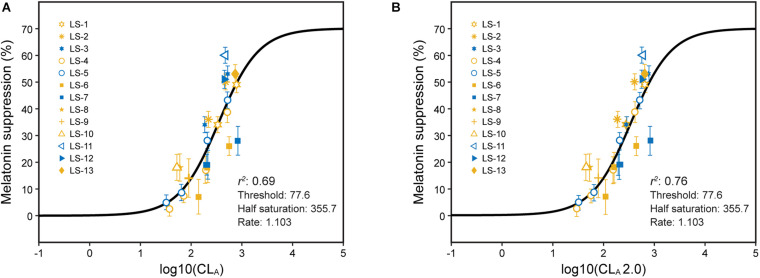
Predictions of absolute melatonin suppression for 1-h exposures to polychromatic sources from Eqs. 1 and 2 **(A)** and from Eqs. 2 and 3 **(B)**. The legend entries correspond to the light source designations in Supplementary Table 1 and Supplementary Figure 1. Blue symbols are associated with light sources where b-y > 0 and yellow symbols for light sources where b-y ≤ 0. It should be noted, as described in Supplementary Table 1, the different experiments used different spatial distributions to deliver the photic stimulus.

Since 2005, the results from a number of experiments aimed specifically at testing the model have been published. Melatonin suppression after 1 h of light exposure during the early biological night was always measured. A summary of these experiments along with various ways to characterize the photic stimulus are provided in Supplementary Table 1. The ability of the 2005 CS model (Eqs. 1 and 2) to predict nocturnal melatonin suppression following 1-h exposures to the different spectra described in Supplementary Table 1 and Supplementary Figure 1 was good, but not perfect, with an overall coefficient of determination, *r*^2^, of 0.69 (Figure 3).

The value of an overall *r*^2^ can belie, however, any potential systematic errors in the model predictions. Over the years, the model has consistently been able to predict nocturnal melatonin suppression of “cool,” polychromatic light sources, but has not been able to accurately predict suppression from “warm” light sources. Nocturnal melatonin suppression was systematically *overestimated* for “warm” LED light sources that produced radiant energy throughout the spectrum in a study by Nagare et al. (2019c) Conversely, a second study by Nagare et al. (2019b) revealed that nocturnal melatonin suppression was systematically *underestimated* for “warm” LED light sources with a discontinuity in spectral energy around 480 nm. The original model used an *in vivo* estimate of ipRGC-melanopsin spectral sensitivity alone to characterize the spectral sensitivity of the circadian phototransduction circuit to “warm” sources, so this simple assumption for determining CL_*A*_ for “warm” sources was clearly inadequate.

The 2005 two-state CL_*A*_ formulation (Eq. 1) did not include a physiologically based threshold term for the ipRGC-melanopsin response. In the revised formulation (CL_*A*_ 2.0, Eq. 3), the ipRGC-melanopsin response is directly modulated by a threshold term involving both rods and cones that, through the AII amacrine neuron, elevates the threshold response of the M1 ipRGCs to light [see companion paper (https://www.frontiersin.org/articles/10.3389/fnins.2020.615305/full) and the 2005 publication (Rea et al., 2005) for details describing the proposed underlying physiology].


$$C\,L_{A}\,2.0=$$

$$1548\{(\int Mc_{\lambda }E_{\lambda }d\lambda -a_{r\,o\,d\,1}\left(\frac{\int V_{\lambda }^{\prime }\,E_{\lambda }\,d\,\lambda }{\int V_{c\,\lambda }\,E_{\lambda }\,d\,\lambda +g_{1}\,\int S_{c\,\lambda }\,E_{\lambda }\,d\,\lambda }\right)$$

$$\left(1-e^{\frac{-\int V_{\lambda }^{\prime }\,E_{\lambda }\,d\,\lambda }{R\,o\,d\,S\,a\,t}}\right))$$

$$+(a_{b-y}\left(\int S_{c\,\lambda }E_{\lambda }d\lambda -k\int V_{c\,\lambda }E_{\lambda }d\lambda \right)-$$

$$a_{r\,o\,d\,2}\left(\frac{\int V_{\lambda }^{\prime }\,E_{\lambda }\,d\,\lambda }{\int V_{c\,\lambda }\,E_{\lambda }\,d\,\lambda +g_{2}\,\int S_{c\,\lambda }\,E_{\lambda }\,d\,\lambda }\right)\left(1-e^{\frac{-\int V_{\lambda }^{\prime }\,E_{\lambda }\,\text{d}\,\lambda }{R\,o\,d\,S\,a\,t}}\right)),\;\;\;\;\;\;b-y>0$$

$$(\int Mc_{\lambda }E_{\lambda }d\lambda -a_{r\,o\,d\,1}\left(\frac{\int V_{\lambda }^{\prime }\,E_{\lambda }\,d\,\lambda }{\int V_{c\,\lambda }\,E_{\lambda }\,d\,\lambda +g_{1}\,\int S_{c\,\lambda }\,E_{\lambda }\,d\,\lambda }\right)$$

(3) $$\left(1-e^{\frac{-\int V_{\lambda }^{\prime }\,E_{\lambda }\,d\,\lambda }{R\,o\,d\,S\,a\,t}}\right)),b-y\leq 0.$$

where,


$$b-y=\int S_{c\,\lambda }\,E_{\lambda }\,\text{d}\,\lambda -k\,\int V_{c\,\lambda }\,E_{\lambda }\,\text{d}\,\lambda$$

**Table:** 

*k* = 0.2616	*E*_λ_: light source spectral irradiance.
*a*_*b*−*y*_ = 0.21	*Mc*_λ_: melanopsin sensitivity (corrected for crystalline lens transmittance) (Wyszecki and Stiles, 1982)
*a*_*rod*1_ = 2.30	*S*_λ_: S-cone fundamental (Smith and Pokorny, 1975).
*a*_*rod*2_ = 1.60	*mp*_λ_: macular pigment transmittance (Snodderly et al., 1984).
*g*_1_ = 1.00	*V*_λ_: photopic luminous efficiency function (Commission Internationale de l’Éclairage, 1994).
*g*_2_ = 0.16	$V_{\lambda }^{\prime }:$ scotopic luminous efficiency function (Commission Internationale de l’Éclairage, 1994).
*RodSat* = 6.50*Wm*^−2^	$V_{c\,\lambda }=\frac{\left(\frac{V_{\lambda }}{m\,p_{\lambda }}\right)}{\text{max}\,\left(\frac{V_{\lambda }}{m\,p_{\lambda }}\right)}\;S_{c\,\lambda }=\frac{\left(\frac{S_{\lambda }}{m\,p_{\lambda }}\right)}{\text{max}\,\left(\frac{S_{\lambda }}{m\,p_{\lambda }}\right)}$

The coefficient of determination was improved for predicting 1-h exposures using Eq. 3 (CL_*A*_ 2.0) rather than Eq. 2 to characterize *circadian light* (*r*^2^ = 0.76; Figure 3B). In particular, the discrepancies between “warm” and “cool” sources were reduced as was the discrepancy between two types of “warm” LED sources, one with a “gap” near 480 nm and one without.

The spatial distribution of the light sources used to test the 2005 model varied. It has been assumed that a cosine spatial sensitivity would be sufficient for characterizing the effectiveness of flux incident on the cornea (Van Derlofske et al., 2002), but this assumption had never been thoroughly explored. It was also important to better understand the impact of light exposure duration, so this variable was also explored. Supplementary Table 1 provides details about the spatial and temporal characteristics of the light sources used in the experiments aimed at testing the 2005 model.

## Duration and Distribution of Light Exposure

The model of circadian phototransduction published in 2005 and the revised model described here represent the “instantaneous” response from, and thus a stimulus to, a single neural circuit in the retina stimulating the SCN. Even though it took a finite amount of time to experimentally determine CL_*A*_ and CS (1 h of exposure), these two quantities can now be taken from the psychological response domain to the physical stimulus domain characterizing the spectral sensitivity of a single circadian phototransduction circuit as well as an important response characteristic to different amounts of spectrally weighted optical radiation (Rea et al., 2020). The model of circadian phototransduction (Eqs. 2 and 3) is, however, silent with respect to different exposure durations and the distributions of light exposures across the retina. Clearly the duration and distribution of light exposure will affect the total amount of melatonin suppressed at night. For a fixed spectrum and amount, exposures of longer durations and covering more area of the retina will produce greater attenuation (and vice versa) (Novotny et al., 2013; Nagare et al., 2019a). Thus, to predict the total amount of nocturnal melatonin suppression from light exposures of different durations and spatial extent, CL_*A*_ and CS must be augmented by other aspects of the luminous stimulus, including, but not limited to, exposure duration and light distribution across the retina.

From a modeling perspective it is important to begin by conceptually separating the spectral and absolute sensitivities of the neural circuit stimulating the SCN from the temporal and spatial dynamics of the SCN-pineal interaction. For example, CS may be able to describe the spectrally weighted amount of circuit stimulation to the SCN from light reaching the retina at any time of day or night, but only during the night will that stimulation have an effect on melatonin synthesis by the pineal (Refinetti, 2006). As another example in the temporal domain, a rapid flickering light with the same total energy impinging on the retina as a steady light will not produce the same levels of melatonin suppression (Figueiro et al., 2013; Najjar and Zeitzer, 2016). Spatially, light entering the eyes from a point source will not be as effective as the same amount of light entering the eyes from an extended source (Novotny et al., 2013). Also, the same amount of light entering one eye as that entering both eyes will not produce equivalent levels of suppression (Spitschan and Cajochen, 2019). In general, CL_*A*_ 2.0 and CS, as measures of photic stimulus to the circadian system, are independent of the temporal dynamics of the phototransduction circuits (a limitation of the model) and are silent with respect to the distribution of these circuits across the retina. Moreover, to predict a given behavioral response like absolute nocturnal melatonin suppression or circadian phase changes, additional factors associated with the downstream physiology must be taken into account.

The primary purpose of the revised model (Eqs. 2 and 3) was to improve the characterization of the circadian phototransduction circuit to different spectra and amounts of optical radiation on the human retina. To validate predictions of the circadian phototransduction model, however, the spatial and temporal characteristics of the luminous stimulus must also be considered because they are always part of the stimulus conditions in an experiment. If after taking into account the temporal and spatial characteristics of the photic stimulus, CL_*A*_ 2.0 and CS can be used successfully to predict nocturnal melatonin suppression, then it is logical to assume that these two quantities characterize the spectral sensitivity and the response characteristic of phototransduction circuits stimulating the SCN.

**FIGURE 4:**
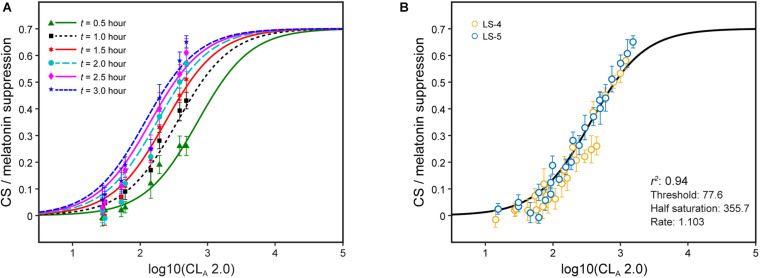
Nocturnal melatonin suppression following different continuous light exposure durations **(A)** and after modifying the half-saturation constant by applying Eq. 2 **(B)**. The legend entries in panel B correspond to the light source designations in Supplementary Table 1 and Supplementary Figure 1; blue symbols are associated with light sources where b-y > 0 and yellow symbols for light sources where b-y ≤ 0.


Recently, we showed that a single factor, *t*, could modulate CL_*A*_ in the CS formulation from the 2005 model (Eqs. 1 and 2) to predict melatonin suppression for different continuous light exposure durations during the early biological night (i.e., just after the expected time of DLMO) (Rea et al., 2020). The absolute suppression values for different continuous light exposure durations are shown along with the revised model predictions (Eqs. 2 and 3) after fixing all of the parameters in the CS formulation except the half-saturation constant (Figure 4A). The half-saturation constants needed to minimize the residual error for each set of data, one for each exposure duration, were determined. These values, plotted as a function of continuous light exposure duration, were fitted with an allometric function, simplified to be consistent with the 2005 model half-saturation constant (i.e., 355.7 based on 1-h exposure), as follows:

(4) $$Half-satconstant2.0=355.7\times t{}^{-1.0}$$

where, *t* serves the purpose of a scalar representing light exposure duration in hours. The coefficient of determination, *r*^2^, for the simplified allometric fit was equal to 0.93.

The single factor, *t*, applied to the half saturation constant from Eq. 2 (355.7) can rectify the different continuous light exposure durations while maintaining all of the parameters in Eq. 2 [It should be noted that the abscissa in Figure 4B is log CL_*A*_ 2.0 (Eq. 3), not CL_*A*_ (Eq. 1) as in the original paper] (Figure 4B). In effect, *t* can simply modify the half saturation constant in Eq. 2 to predict absolute nocturnal melatonin suppression for any continuous light exposure duration from 0.5 to 3 h without any modifications to the CS formulation itself. This being the case, it can be logically inferred that CL_*A*_ 2.0 and CS accurately characterize the spectral sensitivity and the operating characteristics of the modeled circadian phototransduction circuit in the retina.

Following this same logic used to model the duration of light exposure, we explored the possibility that a single parameter, *f*, representing the spatial distribution of circadian light exposure could be used to augment to CS formulation to predict nocturnal melatonin suppression (Eqs. 2 and 3). This initial approach must be inherently of low precision because there is only limited understanding of the anisotropic distribution of circadian phototransduction circuits across the retina (Rea et al., 2020). For modeling purposes then, only three levels of spatial distribution of the luminous stimulus were assumed, (a) full visual field, as with a Ganzfeld, *f* = 2.0 (b) central visual field, as with a discrete light box on a desk, *f* = 1.0 and (c) superior visual field, as from ceiling mounted down-light fixtures, *f* = 0.5. These three levels of *f* were developed to make a relative estimate of how many circadian phototransduction circuits were activated in the four studies used for testing the model and described in Supplementary Table 1.

**FIGURE 5:**
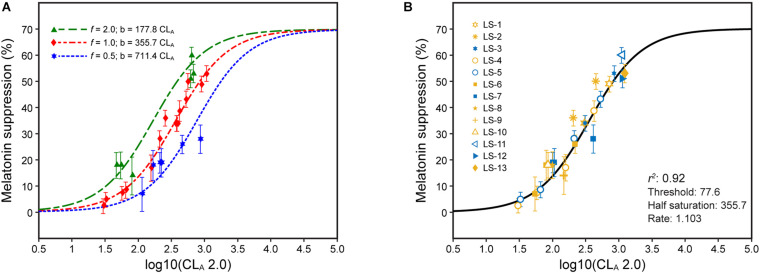
The data in panels **(A,B)** are for 1-h exposures, plotted as a function of CL_*A*_ 2.0 from Eq. 3. **(A)** shows the data separated in terms of three different spatial distributions of the light stimulus: *f* = 2.0 (green), *f* = 1.0 (red), and *f* = 0.5 (blue). **(B)** shows these same data now adjusted for spatial distribution with the solid line reflecting the CS_*t,f*_ formulation of Eq. 5. The legend entries in **(B)** correspond to the light source designations in Supplementary Table 1 and Supplementary Figure 1; blue symbols are associated with light sources where b-y > 0 and yellow symbols for light sources where b-y ≤ 0.


Equation 5 describes the duration- and distribution-augmented CS formulation (Eq. 2), where *t*, the duration factor, is a continuous variable from 0.5 to 3.0 and *f*, the distribution factor, is a discrete variable equal to 2, 1, or 0.5 depending upon the spatial distribution of the light source used in the experiment. The relationships between the revised CL_*A*_ 2.0 (Eq. 3) and melatonin suppression from four experiments along with the distribution-adjusted CS formulation was developed (Figure 5); the duration was 1 h for each of these four sets of data (i.e., *t* = 1.0). Here again, utilizing the distribution form factor *f* to predict absolute nocturnal melatonin suppression supports the inference that CL_*A*_ 2.0 and CS are accurate characterizations of the circadian phototransduction circuit in the retina. For this reason, Eq. 5 can be used to augment the CS formulation (Eq. 2) to predict absolute melatonin suppression for different continuous light exposure durations during the early biological night and different distributions.

(5) $$C\,S_{t,f}=0.7*\left[1-\frac{1}{1+{\left(\frac{t*f*C\,L_{A}}{355.7}\right)}^{1.1026}}\right]$$

## Discussion

**FIGURE 6:**
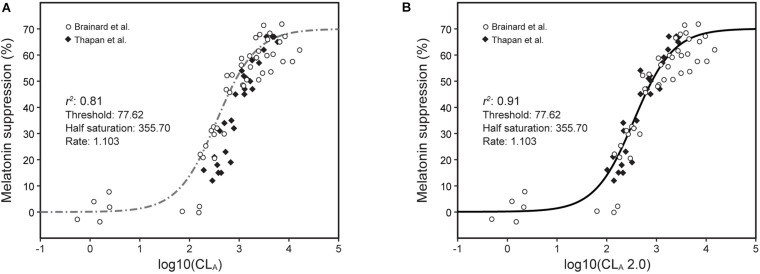
Nocturnal melatonin data for different narrowband light sources from Brainard et al. (2001) (open circles) and Thapan et al. (2001) (solid diamonds) along with predictions from the 2005 CS model (Rea et al., 2005, 2012; Rea and Figueiro, 2018) [Eqs. 1 and 2, dashed line, **(A)**] and from the revised CS_*t,f*_ model [Eqs. 3 and 5, solid line, **(B)**] using the appropriate temporal duration and spatial distribution terms, *t* and *f*, respectively.

**FIGURE 7:**
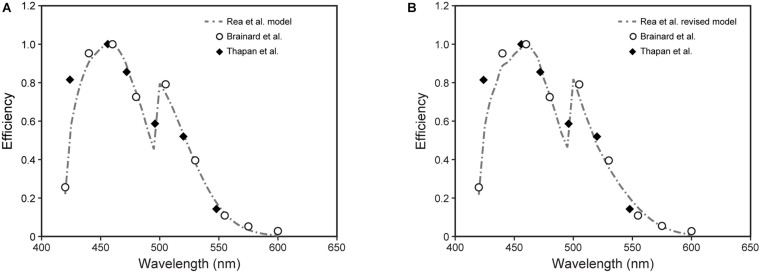
The spectral sensitivities to narrowband light sources from Brainard et al. (2001) (open circles) and Thapan et al. (2001) (solid diamonds) along with predictions from the 2005 (Rea et al., 2005, 2012; Rea and Figueiro, 2018) **(A)** and revised **(B)** CS models (dashed lines).

**FIGURE 8:**
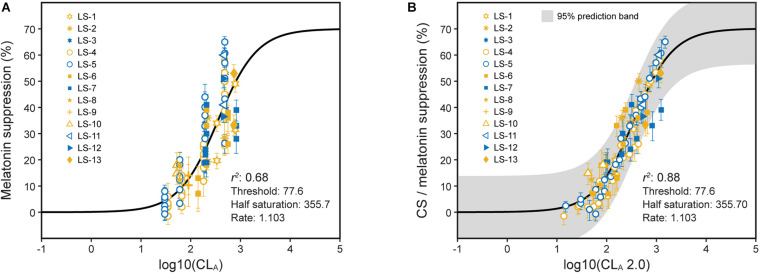
Nocturnal melatonin suppression as a function of log CL_*A*_ **(A)** and log CL_*A*_ 2.0 **(B)**. **(A)** Includes the predictions from the 2005 model (Eqs. 1 and 2, solid line) and **(B)** includes the CS_*t,f*_ predictions from the revised model (Eqs. 3 and 5, solid line). The legend entries in both panels correspond to the light source designations in Supplementary Table 1 and Supplementary Figure 1. Blue symbols are associated with light sources where b-y > 0 and yellow symbols for light sources where b-y ≤ 0.

Direct comparisons between the model predictions from the 2005 and the revised 2020 model were developed (Figures 6–8).

The narrowband data from Brainard et al. (2001) and Thapan et al. (2001) can be modeled using Eq. 5 with and without taking into account the duration of exposure factor, *t* (Figure 6). As can be seen in the left panel of this figure, there are clearly two sets of data, one (open circles) for Brainard et al. (2001) who used 90-min exposures (*t* = 1.5) and the other (solid diamonds) for Thapan et al. (2001) who used 30-min exposures (*t* = 0.5); light stimuli in both experiments were presented in a Ganzfeld (*f* = 2.0). Also plotted are the CS predictions without adjusting for the durations of light exposure (Eq. 1, dot/dash line in Figure 6A) from the 2005 model (*t* = 1, *f* = 1) and the CS_*t,f*_ predictions (Eqs. 3 and 5, solid line in Figure 6B) from the revised model (Thapan et al., *t* = 0.5, *f* = 2.0; and Brainard et al., *t* = 1.5; *f* = 2.0).

The relative spectral sensitivities derived from the original data in Figure 6 were plotted (Figure 7). Shown are the predictions of those relative sensitivities from the 2005 CS model (Rea et al., 2005, 2012; Rea and Figueiro, 2018) (Eqs. 1 and 2; Figure 7A) along with the predictions from the revised model (Eqs. 3 and 5; Figure 7B). As can be readily appreciated, there is very little difference in the modeled spectral sensitivities. It should be noted that we attempted to re-optimize these predictions of spectral sensitivity to narrowband sources using all of the CIE S 026 fundamentals (Commission Internationale de l’Éclairage, 2018, 2020). The predictions were worse. There was little difference between our fundamentals and those published by CIE except for the action spectrum of melanopsin. In 2005 (Rea et al., 2005), and again here, we used the template published by Wyszecki and Stiles (1982) with an *in vivo* (filtered by the crystalline lens) peak sensitivity at 485 nm and a half-maxim sensitivity of 89 nm to characterize the action spectrum of melanopsin. The CIE on the other hand used the template from Govardovskii et al. (2000) with a peak sensitivity of 490 nm and a half-maximum sensitivity of 84 nm. Thus, the Wyszecki and Stiles (1982) template appears to be better than that from Govardovskii et al. (2000) in characterizing the *in vivo* action spectrum of melanopsin. To our knowledge this is the first, albeit indirect, test of the utility of the CIE S 026 melanopic function for modeling the spectral sensitivity of the human circadian system.

The large sample of psychophysical data using polychromatic sources gathered since 2005 were used to test the model (Figure 8; Supplementary Table 1; and Supplementary Figure 1). These data reflect exposures to different spectra and amounts and different durations and spatial distributions. Those data are plotted as a function of log CL_*A*_ and the CS predictions from the 2005 model (Eqs. 1 and 2) (Figure 8A). The same data have also been depicted as a function of log CL_*A*_ 2.0 along with predictions from the revised model (Eqs. 3 and 5), including factors *t* and *f* for duration and spatial distribution, respectively (Figure 8B). As can be readily appreciated by comparing the two panels, significant progress has been made in more accurately and fully characterizing the photic stimulus for the human circadian system. Moreover, because these various data sets can be transformed to follow a function so simply described by Eq. 5 (Figure 8B), the inference that the updated and augmented revised model accurately describes the spectral sensitivity and the operating characteristics of the circadian phototransduction circuit is well supported.

## Limitations of the Model

It is important to call out several limitations of the model that deserve future research.

First, pupil area was not an important consideration in modeling circadian phototransduction. Subjects in the Brainard et al. (2001) and Thapan et al. (2001) studies were exposed to their different stimulus conditions with dilated pupils. This was not the case in the studies cited in Supplementary Table 1; these subjects observed the luminous stimuli with natural pupils. Nevertheless, it was possible to use CL_*A*_ 2.0 and the duration factor, *t*, derived largely from the latter studies, to rectify the two sets of suppression data from Brainard et al. (2001) and Thapan et al. (2001) (Figure 6). Tentatively, this suggests that there is an adaptation mechanism unaccounted for in the model that discounts the absolute flux density on the retina in favor of adjusting the system gain, perhaps to maintain brightness and color constancy by the visual system (Cornsweet, 1970). A systematic look at circadian phototransduction for different pupil sizes, independent of the luminous stimulus, would be important.

Second, the best fitting exponent for *t* in modeling the effects of duration for up to three continuous hours of exposure (Eq. 4) was originally derived to be −0.85 and further simplified to be −1.0, as explained in the recent duration model paper (Rea et al., 2020), and assumed in the current revised model. An exponent greater than −1.0 (e.g., −0.85) suggests some form of habituation of the entire system over extended exposure durations. Perhaps after 4 or 5 h the impact of habituation would be more obvious than it was from the data from studies in Supplementary Table 1; none of those subjects were exposed to the luminous stimuli for more than three continuous hours. Importantly, habituation is defined in terms of reduced sensitivity to post-receptor sensory input. If habituation was evident after 3 h of exposure, it would likely be associated with the SCN, the pineal gland, or both. Gooley et al. (2010) showed evidence of habituation after exposures to narrow band lights of 555 nm and 460 nm for 6.5 h. They attributed the differential change light sensitivity for 555 nm and 460 nm to a change in spectral sensitivity of the circadian transduction circuit, but this seems unlikely because they did not equate the stimuli in terms of circadian-effective light. Specifically, the maximum stimulus magnitudes of the 555 nm and the 460 nm lights were CS = 0.17 and CS = 0.55, respectively (Nagare et al., 2019a). As a classic characteristic of post-receptor neural habituation, the rate of change in response to stimuli of increasing magnitude was greater for stimuli of low intensity (555 nm) than stimuli of high intensity (460 nm) for long exposure durations. In any event, the value of *t* augmenting the revised model is limited to 3 h of continuous exposure, so, clearly, the effects of prolonged exposures to light at night need to be investigated.

Third, the revised model does not take into account the temporal dynamics of nocturnal melatonin suppression. Specifically, there is strong evidence that model cannot predict melatonin suppression from intermittent light exposures (e.g., Gronfier et al. (2004); Najjar and Zeitzer (2016)). Here again, because one can only infer phototransduction mechanisms from downstream measurements (i.e., nocturnal melatonin suppression), it remains uncertain whether observations of hysteresis is a property of the retina, the SCN, or the pineal gland. Similar to habituation, however, hysteresis is a common characteristic of post-receptor mechanisms. Additional research on the temporal dynamics of the circadian system, most likely from electrophysiology, would have to be conducted to resolve this particular issue before accurate predictions of nocturnal melatonin suppression or phase shifting are possible.

The spatial distribution of phototransduction circuits, as it would affect values of *f*, clearly needs further investigation. There is some controversy in the literature with regard to the most sensitive area of the retina to circadian-effective light. Rüger et al. (2005) and Visser et al. (1999), for example, found that the nasal retina is more sensitive to circadian-effective light exposure than the inferior retina as proposed by Glickman et al. (2003). Our own, unpublished, findings suggest that the macular region is more sensitive than the peripheral retina. Understanding the spatial distribution of circadian phototransduction circuits is of particular interest for clinical and architectural applications. For example, should a light box designed to improve circadian entrainment be placed above or to the side of the patient, or are architectural lighting fixtures in the ceiling plane more or less effective than a window in the wall, or is natural daylight exposure more like a Ganzfeld or more like a ceiling fixture? Answers to the types of questions should be addressed through translational research.

In a similar vein, the recent study by Phillips et al. (2019) shows that melatonin suppression at night is affected by light exposures prior to the natural onset of melatonin synthesis in the early evening. Their protocol apparently contributes significantly to inter-individual differences and strongly suggests that the impact of light exposures in the home at night will be dramatically, and quite idiosyncratically, affected by how much and how long a person was exposed to light during the day. Their findings suggest, therefore, that the operating characteristic of the modeled circadian phototransduction circuit offered here depends upon earlier light exposure. As more in-depth knowledge of these individual differences is obtained, revisions to the proposed model can be made to incorporate these new findings.

## Concluding Remark

Finally, as noted in the Introduction, the circadian phototransduction circuit model described here must converge with the known neurophysiology and neuroanatomy of the human retina. The companion paper (https://www.frontiersin.org/articles/10.3389/fnins.2020.615305/full) provides a circuit diagram of the retina along with supporting discussions that makes the proposed model of circadian phototransduction physiologically plausible.

## Data Availability Statement

The original contributions presented in the study are included in the article/Supplementary Material, further inquiries can be directed to the corresponding author.

## Ethics Statement

The studies involving human participants were reviewed and approved by the Institutional Review Board, Rensselaer Polytechnic Institute. The patients/participants provided their written informed consent to participate in this study.

## Author Contributions

MR performed modeling and served as the primary author of the manuscript. RN performed data collection and modeling, prepared the figures and equations, and contributed to the manuscript. MF supervised the data computation and melatonin analysis, and provided leadership in preparation of the manuscript. All authors contributed to the article and approved the submitted version.

## Conflict of Interest

The authors declare that the research was conducted in the absence of any commercial or financial relationships that could be construed as a potential conflict of interest.
