# Corrigendum: Modeling Circadian Phototransduction: Quantitative Predictions of Psychophysical Data

**DOI:** 10.3389/fnins.2022.849800

**Published:** 2022-02-18

**Authors:** Mark S. Rea, Rohan Nagare, Mariana G. Figueiro

**Affiliations:** ^1^Lighting Research Center, Rensselaer Polytechnic Institute, Troy, NY, United States; ^2^Icahn School of Medicine at Mount Sinai, New York, NY, United States

**Keywords:** circadian light, circadian stimulus, phototransduction, melatonin suppression, light at night, non-image forming effects of light

In the original article, there was an error. One of the inherent mathematical assumptions for Equation 3 as published, should be explicitly added to the mathematical formulation. The published terms “V_λ_” and “S_λ_” therefore have been replaced by “V_cλ_” and “S_cλ_” respectively, to convey the implicit normalizations in the corrected equation. The term *k* has also been defined.

A correction has been made to Equation 3.


$$CL_{A}2.0=1548\left\{ \begin{array}{l} \left(\int Mc_{\lambda }E_{\lambda }d\lambda -a_{rod1}\left(\frac{\int V_{\lambda }^{\prime }E_{\lambda }d\lambda }{\int V_{c\lambda }E_{\lambda }d\lambda +g_{1}\int S_{c\lambda }E_{\lambda }d\lambda }\right)\left(1-e^{\frac{-\int V_{\lambda }^{\prime }E_{\lambda }d\lambda }{RodSat}}\right)\right)\\ \;+\left(a_{b-y}(\int S_{c\lambda }E_{\lambda }d\lambda -k\int V_{c\lambda }E_{\lambda }d\lambda \right)-\;a_{rod2}\left(\frac{\int V_{\lambda }^{\prime }E_{\lambda }d\lambda }{\int V_{c\lambda }E_{\lambda }d\lambda +g_{2}\int S_{c\lambda }E_{\lambda }d\lambda }\right)\\ \;\left(1-\;e^{\frac{-\int V_{\lambda }^{\prime }E_{\lambda }d\lambda }{RodSat}})\right),\;b-y>0\;\\ \left(\int Mc_{\lambda }E_{\lambda }d\lambda -a_{rod1}\left(\frac{\int V_{\lambda }^{\prime }E_{\lambda }d\lambda }{\int V_{c\lambda }E_{\lambda }d\lambda +g_{1}\int S_{c\lambda }E_{\lambda }d\lambda }\right)\left(1-e^{\frac{-\int V_{\lambda }^{\prime }E_{\lambda }d\lambda }{RodSat}}\right)\right),\;b-y\leq 0\; \end{array}\right.$$

where,


$$b-y=\int S_{c\lambda }E_{\lambda }d\lambda -k\int V_{c\lambda }E_{\lambda }d\lambda$$

**Table:** 

*k* = 0.2616	*E*_λ_: light source spectral irradiance.
*a*_*b*−*y*_ = 0.21	*Mc*_λ_: melanopsin sensitivity (corrected for crystalline lens spectral transmittance) (Wyszecki and Stiles, 1982)
*a*_*rod*1_ = 2.30	*S*_λ_: S-cone fundamental (Smith and Pokorny, 1975).
*a*_*rod*2_ = 1.60	*mp*_λ_: macular pigment spectral transmittance (Snodderly et al., 1984).
*g*_1_ = 1.00	*V*_λ_: photopic luminous efficiency function (Commission Internationale de l'Éclairage, 1994).
*g*_2_ = 0.16	${V^{\prime }}_{\lambda }$: scotopic luminous efficiency function (Commission Internationale de l'Éclairage, 1994).

*RodSat* = 6.50 *W m*^−2^


$$\begin{array}{l} V_{c\lambda }=\frac{\left(\frac{V_{\lambda }}{mp_{\lambda }}\right)}{\max\left(\frac{V_{\lambda }}{mp_{\lambda }}\right)}\;S_{c\lambda }=\;\frac{\left(\frac{S_{\lambda }}{mp_{\lambda }}\right)}{\max\left(\frac{S_{\lambda }}{mp_{\lambda }}\right)} \end{array}$$

The authors apologize for this error and state that this does not change the scientific conclusions of the article in any way. The original article has been updated.

## Publisher's Note

All claims expressed in this article are solely those of the authors and do not necessarily represent those of their affiliated organizations, or those of the publisher, the editors and the reviewers. Any product that may be evaluated in this article, or claim that may be made by its manufacturer, is not guaranteed or endorsed by the publisher.

