# Supplementary material for: Modeling Circadian Phototransduction: Quantitative Predictions of Psychophysical Data
Source: Front Neurosci. 2021 Feb 5;15:615322. doi: 10.3389/fnins.2021.615322 (PMC7893103; doi:10.3389/fnins.2021.615322)
Supplement: Supplementary file 1 [file Data_Sheet_1.PDF]

Modeling circadian phototransduction: Quantitative predictions of psychophysical data

Mark S. Rea [1,2]*, Rohan Nagare [1,2], Mariana G. Figueiro [1,2]

[1] Lighting Research Center, Rensselaer Polytechnic Institute, Troy, NY, 12180, United States

[2] Icahn School of Medicine, Mount Sinai, New York, NY, 10029, United States

* To whom all correspondence should be addressed: Mark S. Rea, Lighting Research Center, 21 Union Street, Troy, NY 12180-3352, USA; e-mail: mark.rea@mountsinai.org

Supplementary Material

Supplementary Table S1

Supplementary Figure S1

Supplementary Table S1. Data included in the modeling exercise while developing the revised model, as well as α-opic irradiances, calculated from the Commission Internationale de l'Éclairage (Commission Internationale de l'Éclairage, 2018;2020) for the data included in the modeling exercise.
The parameters f and t (experimental design column) represent the spatial distribution of the light source and the duration of light exposure, respectively, used in each experiment.

Data set	Experimental design	LED source	CCT (K)	Photopic Illuminance (lx)	CS (1-h)	Melatonin suppression (1 hour)	S-cone-opic irradiance $W m^{-2}$	M-cone-opic irradiance $W m^{-2}$	L-cone-opic irradiance $W m^{-2}$	Rhodopic irradiance $W m^{-2}$	Melanopic irradiance $W m^{-2}$
Nagare et al. (2019b)	Melatonin suppression was determined using 16 adult participants exposed to linear on-axis light bars (*f* = 1.0) for an hour (00:00 – 01:00; *t* = 0.5, 1.0) to three white light sources (3000 K, 3000 K cyan-gap, 4000 K) at two photopic light levels each (≈ 400 lx & 800 lx)	LS-1a	3000	384	0.34	0.34	0.10	0.44	0.64	0.28	0.22
		LS-1b		898	0.49	0.49	0.24	1.03	1.49	0.66	0.51
		LS-2a	3000 cyan-gap	361	0.26	0.36	0.13	0.41	0.58	0.20	0.13
		LS-2b		787	0.41	0.50	0.28	0.90	1.27	0.44	0.29
		LS-3a	4000	361	0.23	0.34	0.15	0.45	0.59	0.34	0.27
		LS-3b		966	0.43	0.53	0.40	1.21	1.57	0.90	0.73
Nagare et al. (2019c)	Melatonin suppression was determined using 23 adults and 18 adolescent participants exposed to linear on-axis light bars (*f* = 1.0) for three hours (23:00 – 02:00; *t* = 0.5 – 3.0) to two white light sources (2700 K, 6500 K) at four photopic light levels each (≈ 40 lx – 1000 lx)	LS-4a	2700	55	0.06	0.03	0.01	0.06	0.09	0.04	0.03
		LS-4b		105	0.12	0.07	0.02	0.12	0.17	0.08	0.06
		LS-4c		286	0.27	0.17	0.06	0.32	0.47	0.21	0.15
		LS-4d		760	0.45	0.39	0.16	0.85	1.26	0.56	0.41
		LS-5a	6500	32	0.06	0.05	0.02	0.04	0.05	0.04	0.04
		LS-5b		64	0.11	0.09	0.05	0.09	0.10	0.08	0.07
		LS-5c		204	0.28	0.28	0.15	0.28	0.33	0.26	0.22
		LS-5d		502	0.45	0.43	0.38	0.70	0.80	0.63	0.55
Nagare et al. (2019a)	Melatonin suppression was determined using 12 adults and 12 adolescent participants exposed to two overhead ceiling (*f* = 0.5) white light fixtures (2700 K, 5600 K) for four hours (23:00 – 03:00 *t* = 0.5 – 3.0) at three light levels each (≈ 200 lx, 800 lx and CS of 0.25)	LS-6a	2700	200	0.19	0.07	0.04	0.22	0.33	0.13	0.09
		LS-6b		295	0.44	0.26	0.05	0.32	0.49	0.19	0.14
		LS-6c		800	0.25	0.18	0.14	0.87	1.34	0.51	0.37
		LS-7a	5600	200	0.24	0.19	0.13	0.27	0.32	0.23	0.20
		LS-7b		210	0.50	0.28	0.14	0.29	0.34	0.24	0.21
		LS-7c		800	0.25	0.19	0.52	1.09	1.29	0.93	0.80
LRC (unpublished)	Melatonin suppression was determined using 14 adult participants exposed for an hour (00:00 – 01:00; *t* = 0.5, 1.0) via diffused light boxes (*f* = 2.0) to three low-CCT white light sources (≈ 100 lx) and three high-CCT white light sources (≈ 500 lx)	LS-8	2200	100	0.09	0.18	0.01	0.11	0.17	0.06	0.04
		LS-9	2700	100	0.13	0.14	0.02	0.12	0.17	0.08	0.06
		LS-10	2600	100	0.08	0.18	0.02	0.11	0.16	0.06	0.04
		LS-11	4700	500	0.41	0.60	0.28	0.68	0.81	0.61	0.53
		LS-12	4900	500	0.40	0.51	0.27	0.69	0.81	0.61	0.53
		LS-13	3200	500	0.49	0.53	0.17	0.63	0.84	0.55	0.49

<table>
<tr><th>Data set</th><th>Relative spectral power distributions</th></tr>
<tr><td>Nagare et al. (2019b)</td><td>
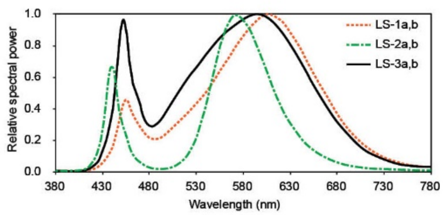

</td></tr>
<tr><td>Nagare et al. (2019c)</td><td>
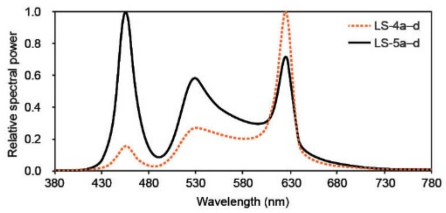

</td></tr>
<tr><td>Nagare et al. (2019a)
(partially published)</td><td>
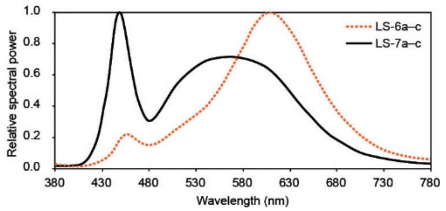

</td></tr>
<tr><td rowspan="2">LRC (unpublished)</td><td>
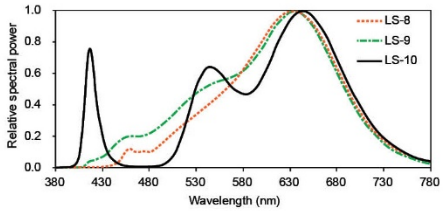

</td></tr>
<tr><td>
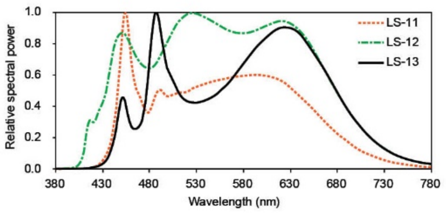

</td></tr>
</table>

Supplementary Figure S1. *Relative spectral power distributions for the light sources used to develop the revised model.*

References Cited

Commission Internationale De L'éclairage (2018). CIE system for metrology of optical radiation for ipRGC-influenced responses to light. Standard CIE S 026/E:2018. (Vienna: Commission Internationale de l'Éclairage).

Commission Internationale De L'éclairage (2020). CIE S 026 α-opic Toolbox - v1.049 - 2020/03/26. (Vienna: Commission Internationale de l'Éclairage). Available for download at https://bit.ly/33YM39Rh

Nagare, R., Plitnick, B., and Figueiro, M.G. (2019a). Effect of exposure duration and light spectra on nighttime melatonin suppression in adolescents and adults. *Lighting Res. Technol.* 51**,** 530-540. doi: 10.1177/1477153518763003

Nagare, R., Rea, M.S., Plitnick, B., and Figueiro, M.G. (2019b). Effect of white light devoid of "cyan" spectrum radiation on nighttime melatonin suppression over a 1-h exposure duration. *J. Biol. Rhythms* 34**,** 195-204. doi: 10.1177/0748730419830013

Nagare, R., Rea, M.S., Plitnick, B., and Figueiro, M.G. (2019c). Nocturnal melatonin suppression by adolescents and adults for different levels, spectra, and durations of light exposure. *J. Biol. Rhythms* 34**,** 178-194. doi: 10.1177/0748730419828056
